# Synthesis and antimicrobial activities of chitosan/polypropylene carbonate-based nanoparticles†

**DOI:** 10.1039/d0ra09257f

**Published:** 2021-03-10

**Authors:** Zhilong Quan, Chunyang Luo, Bitong Zhu, Chungui Zhao, Mingyi Yang, Magnar Bjørås, Kaizheng Zhu, Anna-Lena Kjøniksen

**Affiliations:** College of Materials Science & Engineering, Huaqiao University Xiamen 361021 P. R. China zlquan@hqu.edu.cn; College of Chemical Engineering, Huaqiao University 361021 Xiamen P. R. China; Department of Microbiology, Oslo University Hospital P.O. Box 4950 N-0424 Oslo Norway; Faculty of Engineering, Østfold University of College P.O. Box 700 N-1757 Halden Norway anna.l.kjoniksen@hiof.no

## Abstract

Antibiotic resistance is an emerging threat to public health. The development of a new generation of antimicrobial compounds is therefore currently required. Here we report a novel antimicrobial polymer of chitosan/polypropylene carbonate nanoparticles (CS/PPC NPs). These were designed and synthesized from readily available chitosan and a reactive oligomer polypropylene carbonate (PPC)-derived epoxy intermediate. By employing a simple and efficient functionalized strategy, a series of micelle-like chitosan-*graft*-polypropylene carbonate (CS-*g*-PPC) polymers and chitosan–polypropylene carbonate (CS–PPC) microgels were prepared by reacting mono-/bis-epoxy capped PPC with chitosan. The chemical structure, particle size, and surface charge of the newly synthesized polymers were characterized by infrared (IR) spectroscopy, nuclear magnetic resonance (NMR) spectroscopy, dynamic light scattering (DLS), and zeta potential measurements. The antimicrobial activities of these nanoparticles were determined in both Gram-positive bacteria (*S. aureus*) and Gram-negative bacteria (*E. coli*). Minimum inhibitory concentration (MIC), the nanoparticle concentration needed to completely inhibit the bacterial growth, was found at 128 μg mL^−1^ to 1024 μg mL^−1^, strongly depending both on the nature of the epoxy-imine network formed from the functional groups (mono- or bis-capped epoxy groups reacting with amine groups) and the feed ratio of the functional groups (-epoxy/-NH_2_) between the functionalized PPC and chitosan. No hemolysis was observed at concentrations well in excess of the effective bacteria-inhibiting concentrations. These findings provide a novel strategy to fabricate a new type of nanoantibiotic for antimicrobial applications.

## Abstract 2

Chitosan-*graft*-polypropylene carbonate polymers (M-type) and microgels (G-type) exhibit high antimicrobial activity against *E. coli* and *S. aureus* and display no hemolytic activity towards mouse red blood cells in neutral medium.

## Graphical abstract

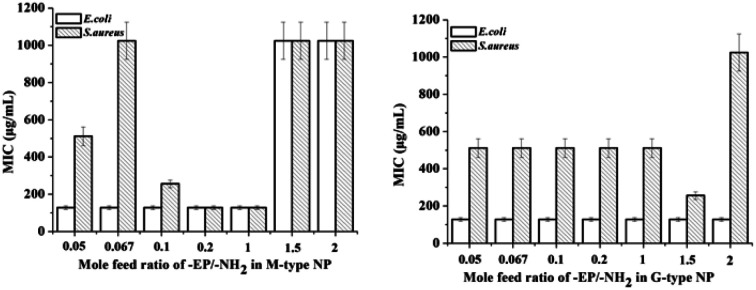

## 1. Introduction

Antibiotic resistance of life-threatening bacteria is a major threat to public health.^1^ Nearly a fifth of infections occurring in wealthy countries (Europe, North America, and Australia) are due to antibiotic resistant bacteria.^2^ Estimations predict 10 million annual deaths due to antimicrobial resistance by 2050, which will surpass the number of deaths caused by cancer.^3^ Scientists are looking for new anti-bacterial materials which are non-toxic, environmentally friendly, exhibit a potent and broad-range antimicrobial activity with a long-lasting response, and are reusable while maintaining their activity. The combination of material science and nanomedicine has resulted in a new alternative field that involves functionalizing nanostructures with several biologically active materials.^4^ Due to their small size, nanomaterials exhibit unique properties that can be leveraged to improve upon the sensitivity, specificity, throughput, and potency of current modalities. Nanoparticles can be tailor-made for antimicrobial applications.^5,6^ Preparation of antimicrobial nanoparticles (NPs) is potentially cost-effective compared to antibiotic synthesis, and the NPs can be stable for long-term storage with a prolonged shelf-life.^7^

Chitosan is a linear polysaccharide copolymer consisting of β-1,4-linked d-glucosamine and *N*-acetyl-glucosamine, which is obtained by partial alkaline *N*-deacetylation of chitin.^8^ Chitosan has recently attracted attention due to its significant antimicrobial properties and the advantages of being nontoxic, biodegradable and biocompatible.^9,10^ A number of commercial applications of chitosan benefit from its antimicrobial activity, including its use in food preservation, in dentistry and ophthalmology, as well as in the manufacture of wound-dressings, and antimicrobial-finished textiles.^11,12^ However, the antimicrobial properties of chitosan are mostly limited to pH below p*K*_a_ (about 6.5), since chitosan begins to lose its cationic properties and its solubility at higher pH values. This can limit its applications at physiological conditions. Overcoming these difficulties by improving the solubility of chitosan derivatives over a wider pH range is therefore necessary. Chitosan contains of three types of nucleophilic functional groups: a C-2 NH_2_ group, a C-3 secondary OH group, and a C-6 primary OH group. Chitosan can be chemically modified either at the amino group or at the hydroxyl groups to produce derivatives containing cationic or other hydrophilic or hydrophobic moieties.^13–15^

Aliphatic polycarbonates (APCs) are a relatively new class of synthetic biomaterials, which are interesting due to their biocompatibility, nontoxic biodegradation products, facile synthesis, and the ease of tailoring the functionalities to meet specific requirements.^16^ APCs exhibit a great potential for antimicrobial applications.^17^ In addition, polycarbonate degradation will not lead to increased levels of acidity, which may occur during polyester degradation, and which may be hazardous to both the loaded drugs and the surrounding tissue.^18^ APCs are approved for application in biological fields by the United States Food and Drug Administration (FDA).^19^ Polypropylene carbonate (PPC) is a fully biodegradable and environment-friendly aliphatic polycarbonate synthesized from carbon dioxide and propylene oxide.^20^ PPC has already been commercialized in spite of the low rigidity and *T*_g_ (from 25 to 45 °C). PPC plants with a total capacity of 50 000 ton per year were commercialized by Boda Dongfang Group Co. in Jinlin, China.^21^

Chitosan nanoparticles can be prepared by means of “physical” or “chemical” crosslinking. For example, chitosan nanoparticles can easily be prepared by the incorporation of polyanions such as tripolyphosphate (TPP) into chitosan solutions under continuous stirring.^22–24^ Chitosan/TPP nanoparticles have been extensively investigated for drug, protein, and gene delivery.^25,26^ However, compared to covalent chemical bonds, physically crosslinked chitosan nanoparticles are characterized by looser networks with lower mechanical properties. The crosslinking process is dependent on various parameters, such as temperature, pH, and ionic strength, or by the addition of suitable counterions. For chemical crosslinking, covalent bonds are utilized to form a permanent polymer network within the nanoparticles. Small molecules such as glutaraldehyde, diglycidyl ether, diisocyanate or diacrylate are typically used to prepare chemically crosslinked nanoparticles of chitosan.^27^ However, the well-known toxicity of most of these molecules represents a serious concern.

Chitosan derivatives have been found to lose most of their antibacterial activity if they are dissolved under neutral conditions. However, chitosan/dextran hydrogels appeared to be antimicrobial in a preliminary work utilizing animal models.^28^

The aim of the present study is to explore a simple synthesis method for a new type of antibacterial nanoparticles combining the biopolymer-chitosan (CS) with a commercially available biodegradable synthetic aliphatic polycarbonate. Epoxy-terminated aliphatic polypropylene polycarbonates (PPC) were employed as the reactive chemical crosslinking agent/grafter. In addition, PPC also acts as hydrophobic units. To the best of our knowledge, CS/PPC nanoparticles have not been previously synthesized. In addition, their utilization as intrinsic antimicrobial materials at neutral pH is new. The CS/PPC nanoparticles were tested for activity against both Gram-positive and Gram-negative bacteria by using the broth microdilution method. These nanoparticles were also tested for hemolytic activities against red blood cells, and the selectivity against bacteria was evaluated.

## 2. Experimental

### 2.1. Materials

Chitosan with a 90% deacetylation degree and a viscosity of 8 mPa s (1%, 20 °C) was purchased from Weifang Haizhiyuan Biological Product Co., Ltd., (Shandong, China). Polypropylene carbonate diol (HO-PPC-OH, *M*_n_ = 2500) was provided by Dazhi Environmental Protection Technology Co., Ltd. (Guangdong, China), Sodium hydride (60% dispersion in mineral oil), toluene and (±)-epichlorohydrin were purchased from Sinopharm Chemical Reagent Co., Ltd (China). Yeast, peptone and agar were purchased from Aladdin Reagent Co., Ltd. (Shanghai, China). The chemicals were used directly without further purification.

### 2.2. Microorganisms

*Escherichia coli* (*E. coli*) and *Staphylococcus aureus* (*S. aureus*) utilized in these studies were purchased from China General Microbiological Culture Collection Center (Beijing, China). 2% mouse red blood cells (RBC) was provided by CenXi Hongquan Bio-technology Co., Ltd (Guangxi, China).

### 2.3. Synthesis

#### 2.3.1. Synthesis of mono- and bis-epoxy-capped polypropylene carbonate (EP-PPC-OH and EP-PPC-EP)

**Scheme 1:**
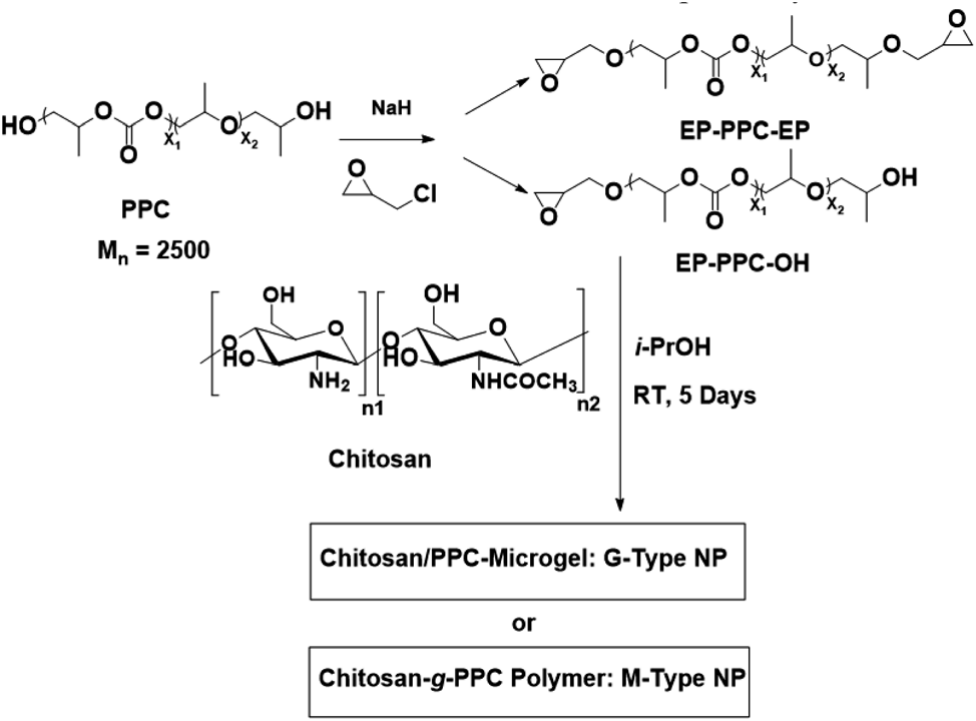
Preparation route of chitosan/polypropylene carbonate (CS/PPC) nanoparticles.

Mono- and bis-epoxy-capped polypropylene carbonates (PPC) were synthesized *via* a simple ‘Williamson ether synthesis’ method involving the commercial polypropylene carbonate diol and (±)-epichlorohydrin, activated by NaH as described in Scheme 1.^29^ PPC (20 g, 8 mmol) was dissolved in toluene (100 mL) and the solvent was azeotropically distilled in the flask to a final volume of 30 mL to remove the residual water adsorbed by the polymer. The flask was allowed to cool to room temperature before sodium hydride was added to the mixture at a molar [NaH]/[PPC] ratio of 1.2 : 1 or 2.4 : 1 for the mono- or bis-products, respectively. After the NaH addition, the reaction mixture was stirred for another 3 h. Epichlorohydrin (at the same molar feed ratio as NaH) was added, after which the solution was stirred at room temperature for another 12 h. The resulting mixture was filtered and extracted with dichloromethane. The organic phase was subsequently washed with water, dried over anhydrous Na_2_SO_4_, concentrated and dried *in vacuo* to achieve the final desired mono- and bis-epoxy capped PPC with the yield of 85% and 92%, respectively. The mono- and bis-epoxy capped PPC are abbreviated as EP-PPC-OH and EP-PPC-EP, respectively.

#### 2.3.2. General procedure for the synthesis of chitosan-*graft*-polypropylene carbonate (CS-*g*-PPC) polymer

Mono-epoxy-capped PPC (EP-PPC-OH) (1.79 g, 0.7 mmol) in i-propyl alcohol (5 mL) was added dropwise to a 1.0 wt% aqueous chitosan solution (12.5 mL). The reaction mixture was stirred for 5 days at room temperature. The resulting chitosan-*g*-PPC was purified by dialyzing against distilled water for 2 weeks using a dialysis membrane of regenerated cellulose with a molecular weight cut-off of 1000, and finally collected by lyophilization. A series of chitosan-*g*-PPC have been synthesized by adjusting the molar feed ratio of EP-PPC/-NH_2_ groups of chitosan ([-EP]/[-NH_2_]) from 0.05 to 2 and they were labeled as M1 to M7 (Scheme 1 and Table 1).

**Table 1:** Biological activity of the nanoparticles

Sample (-EP/-NH_2_)	MIC (μg mL^−1^)		MBC (μg mL^−1^)		HC_50_ (μg mL^−1^)	Selectivity HC_50_/MIC	
	*E. coli*	*S. aureus*	*E. coli*	*S. aureus*		*E. coli*	*S. aureus*
M1 (0.05)	128	512	1024	>1024	2048	16	4
M2 (0.067)	128	1024	1024	>1024	2048	16	2
M3 (0.1)	128	256	512	1024	2048	16	8
M4 (0.2)	128	128	512	1024	2048	16	16
M5 (1.0)	128	128	256	512	2048	16	16
M6 (1.5)	1024	1024	>1024	>1024	2048	2	1
M7 (2.0)	1024	1024	>1024	>1024	2048	2	1
G1 (0.01)	128	512	256	1024	2048	16	4
G2 (0.067)	128	512	256	1024	2048	16	4
G3 (0.1)	128	512	256	1024	2048	16	4
G4 (0.2)	128	512	256	1024	2048	16	4
G5 (1.0)	128	512	256	1024	2048	16	4
G6 (1.5)	128	256	256	512	2048	16	8
G7 (2.0)	128	1024	512	>1024	2048	16	2

#### 2.3.3. General procedure for the synthesis of chitosan–polypropylene carbonate (CS–PPC) microgels

A similar reaction procedure as for CS-*g*-PPC was followed where bis-epoxy-capped PPC (EP-PPC-EP) was employed instead of mono-epoxy-capped PPC (EP-PPC-OH). A series of CS-PPC microgels was synthesized by adjusting the molar feed ratio of EP-PPC (EP-PPC-EP)/-NH_2_ of chitosan ([-EP]/[-NH_2_]) from 0.01 to 2 and they were labeled as G1 to G7 (Scheme 1 and Table 1).

### 2.4. Characterization

Fourier transform infrared spectroscopy (FT-IR) were recorded on a Nicolet™ iS50 ATR-FTIR Spectrometer (Thermo Electron Corporation, USA) using KBr pellets in the region from 400–4000 nm^−1^ at a resolution of 2 cm^−1^ with 32 number of scans. NMR spectra were recorded using a Bruker Avance III HD (500 MHz) in CDCl_3_-*d* or D_2_O. The critical micelle concentration (CMC) of the polymeric micelles was determined by a fluorescence method based on solubilization of water-insoluble fluorescent dye (pyrene) into polymer particles in triple distilled water. Excitation spectra (*λ*_em_ = 393 nm) of pyrene were measured at various polymer concentrations using an Agilent Cary Elipse Fluorescence Spectrophotometer G9800A. The CMC of the polymeric micelles was determined using the intensity ratio of the 330–340 nm band of pyrene in the excitation spectra. The utilized excitation and emission bandwidths were 5 and 2.5 nm, respectively. The *I*_338_/*I*_334_ intensity ratio of the pyrene excitation spectra was plotted against polymer concentration (log[C]), and the intersection of the two tangents was considered as the CMC. Particle sizes were measured by Brookhaven Omni Multi-Angle Particle Size and High Sensitivity Zeta Potential Analyzer (633 nm, 25 °C, scattering angle 173°; sample preparation: 1 mg mL^−1^ dissolved in distilled water and filtered with 0.45 μm filter before measurement). UV-vis spectra were recorded using an A390 Spectrophotometer (Aoyi Instrument).

### 2.5. Antibacterial activities of CS/PPC nanoparticles against *E. coli* and *S. aureus*

#### 2.5.1. Minimal inhibitory concentration (MIC)

The minimal inhibitory concentrations (MIC) of CS/PPC nanoparticles were determined by tube dilution (turbidimetric) method which is based on impeding the augmentation of a microbial culture in a uniform antibiotic solution in a medium that favors rapid growth in the absence of the antibiotic.^30^ Briefly, a 2-fold dilution series of CS/PPC nanoparticle solutions were prepared from a pre-formulated stock solution (2048 μg mL^−1^) (1024, 512, 256, 128, 64, 32, 16, … μg mL^−1^), in a sterile nutrient broth in test tubes. The tubes were inoculated under aseptic conditions with a 20 μL suspension of the freshly prepared bacteria (*E. coli* or *S. aureus*). The positive control was given without polymer and a negative control without bacteria was included. After mixing, the tubes were incubated at 37 °C for 24 h and thereafter examined for growth or turbidity. The minimum concentration that can be supplied in the tubes without cell growth is considered as MIC.

#### 2.5.2. Bacterial concentration (MBC) determination

The minimum bactericidal concentration (MBC), or the lowest concentration of nanoparticles that kills 99.9% of bacteria, was determined by assaying the live organisms in the tubes from the MIC test that showed no growth.^31^ The culture from each well of the micro-broth assay was picked up with loop and sub-cultured on agar plates after 24 hours of incubation. Agar plates were further incubated for 24 hours. The lowest concentration of extracts which exhibited no bacterial growth was considered as MBC. The experiments were repeated in triplicate for each strain. Both the tubes and agar plates were examined for growth or turbidity using unaided eye.

### 2.6. Hemolysis studies (HC_50_) of CS/PPC nanoparticles

Hemolysis was measured following a method with minimum modification.^32^ A 2-fold dilution series of polymer/nanoparticles in saline solution was prepared (2048, 1024, 512, 256, 128, … μg mL^−1^) and kept at 37 °C. 1 mL 2% red blood cell (RBC) suspension was mixed with 1 mL polymer/nanoparticle solution in each tube and incubated for 1 h at 37 °C in an inoculation shaker bath. After the incubation, the suspensions were centrifuged at 4000 rpm for 5 min. The centrifuge supernatant samples were transferred and diluted with the same volume of saline solution, and hemolytic activity was calculated by measuring absorbance at 575 nm using a UV-vis spectrophotometer (A390, Aoyi Instrument) for the determination of released amount of hemoglobin. A saline solution was used as a negative hemolysis control, and distilled water was used as a positive control. The hemolysis % ratio was calculated as:Hemolysis ratio (%) = [(*A*_sample_ − *A*_negative_)/(*A*_positive_ − *A*_negative_)] × 100where *A*_sample_, *A*_positive_, and *A*_negative_ are the absorbance of the polymers/nanoparticles samples, containing blood solution, 2% red blood cells in saline solution (0.9% NaCl), and 2% red blood cells in distilled water, respectively. All data were obtained from the mean value of three replicates.

## 3. Results and discussion

### 3.1. Synthesis of CS-*g*-PPC polymer (M-type) and CS-PPC microgels (G-type)

**Scheme 2:**
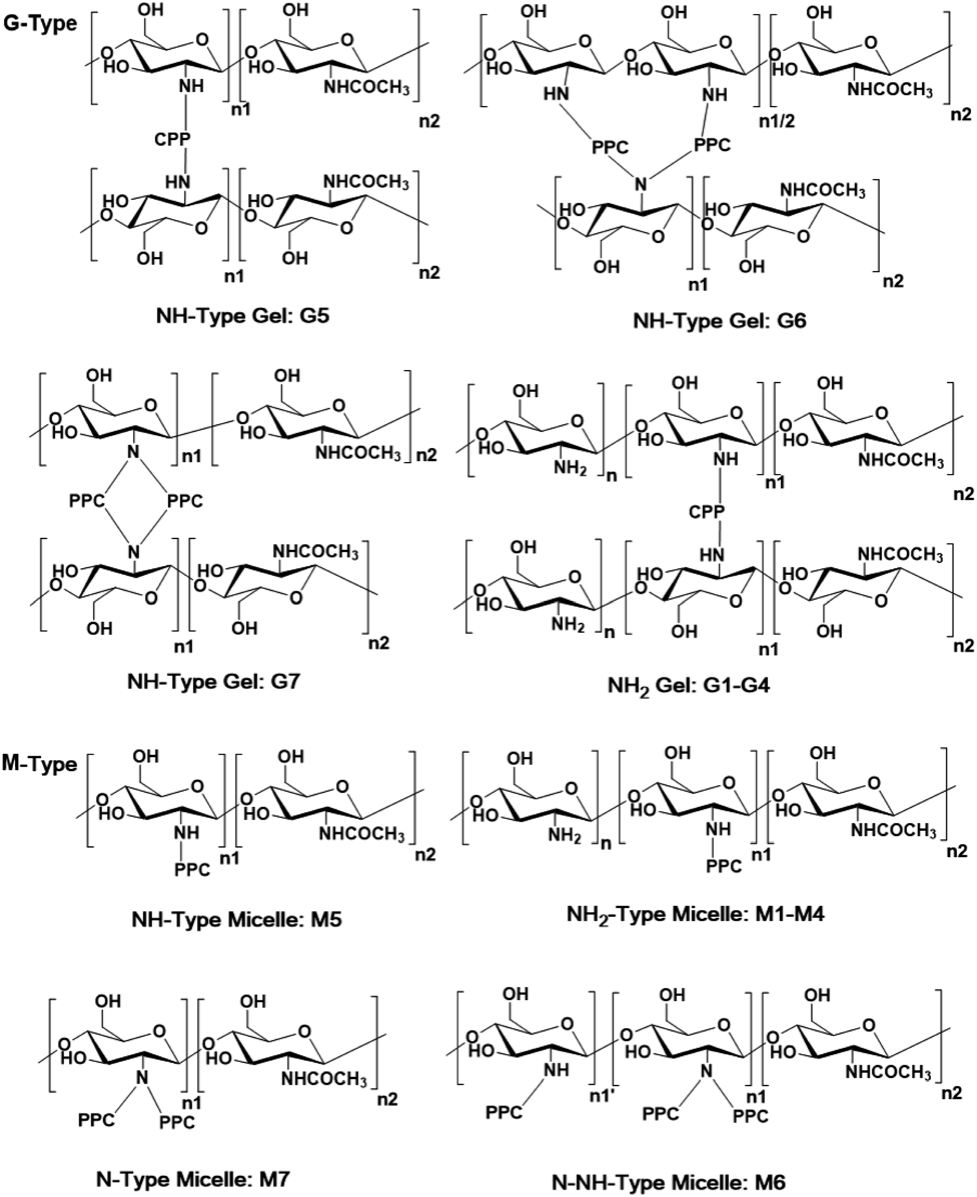
Proposed chemical structures of synthesized G-type and M-type CS/PPC nanoparticles with different feed ratios of CS and EP-PPC-EP (crosslinker) or EP-PPC-OH (grafter).

The synthesis of the antimicrobial CS/PPC nanoparticles consists of two primary steps (Scheme 1): (i) synthesis of the mono- and bis-epoxy capped PPC *via* a “Williamson ether synthesis” by reaction of commercial polypropylene carbonate (PPC) diols with epichlorohydrin; (ii) the resulting mono- and bis-epoxy capped groups of PPC were further reacted with CS to generate the epoxy-imine network of chitosan/polypropylene carbonate (CS/PPC) nanoparticles. CS-*g*-PPC polymers (M-type) and CS–PPC microgels (G-type) (Scheme 2) was achieved by adjusting the feed ratio of EP-PPC/chitosan ([-EP]/[-NH_2_]). Hydroxyalkyl derivatives of chitosan are commonly prepared by treating chitosan with epoxide under heating. In some studies, the epoxide is generated *in situ* under alkaline conditions, after which it undergoes a nucleophilic substitution reaction with the 2-amino or 6-hydroxyl group of chitosan.^33^ However, since the reactions in the current work are performed under very mild conditions (room temperature in the absence of any acid/base catalyst), we hypothesize that the reactions occur between the 2-amino group (the most active group) of chitosan and the epoxy group of PPC. This hypothesis was further confirmed in subsequent NMR and FTIR spectra analysis. This synthetic strategy enables the preparation of new type of antimicrobial nanoparticles in a facile, efficient procedure under very mild reaction conditions.

### 3.2. Chemical structure analysis

**Fig. 1:**
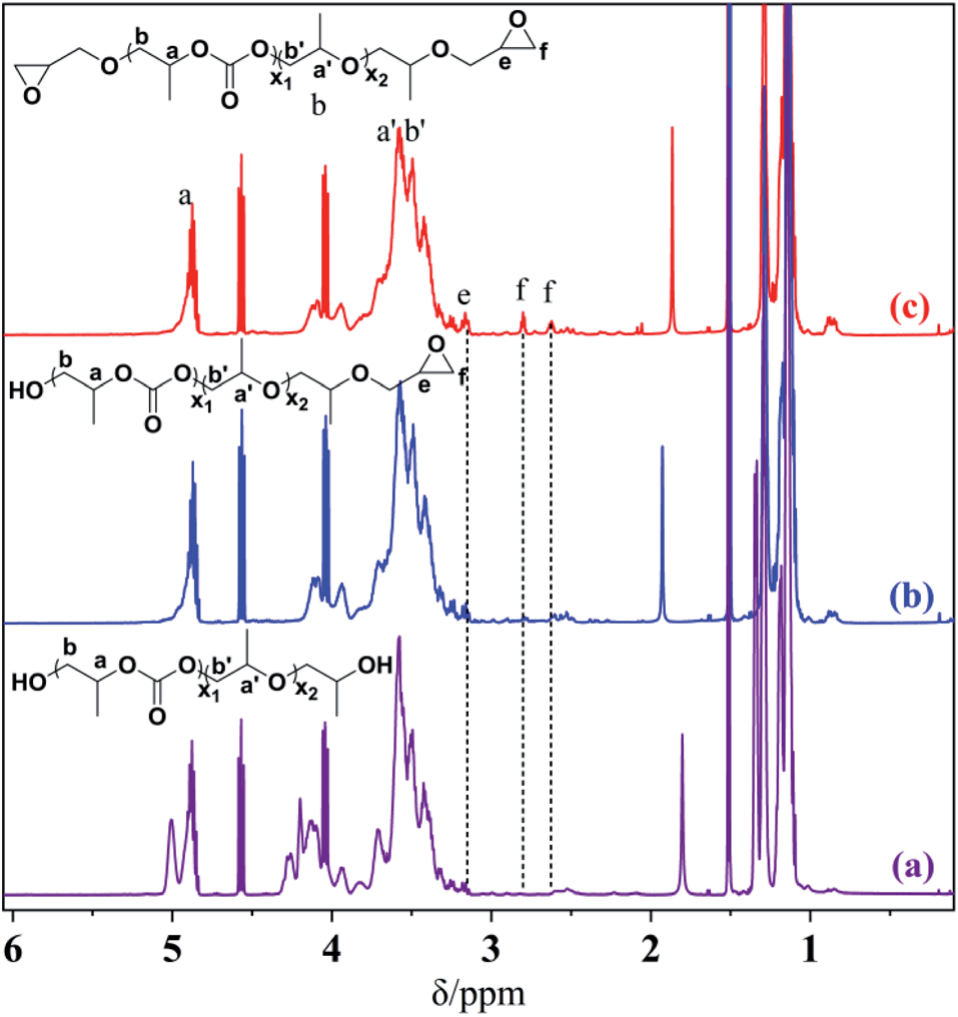
^1^H NMR spectra of HO-PPC-OH (a), EP-PPC-OH (b) and EP-PPC-EP (c) (in CDCl_3_-*d*, 500 MHz).

**Fig. 2:**
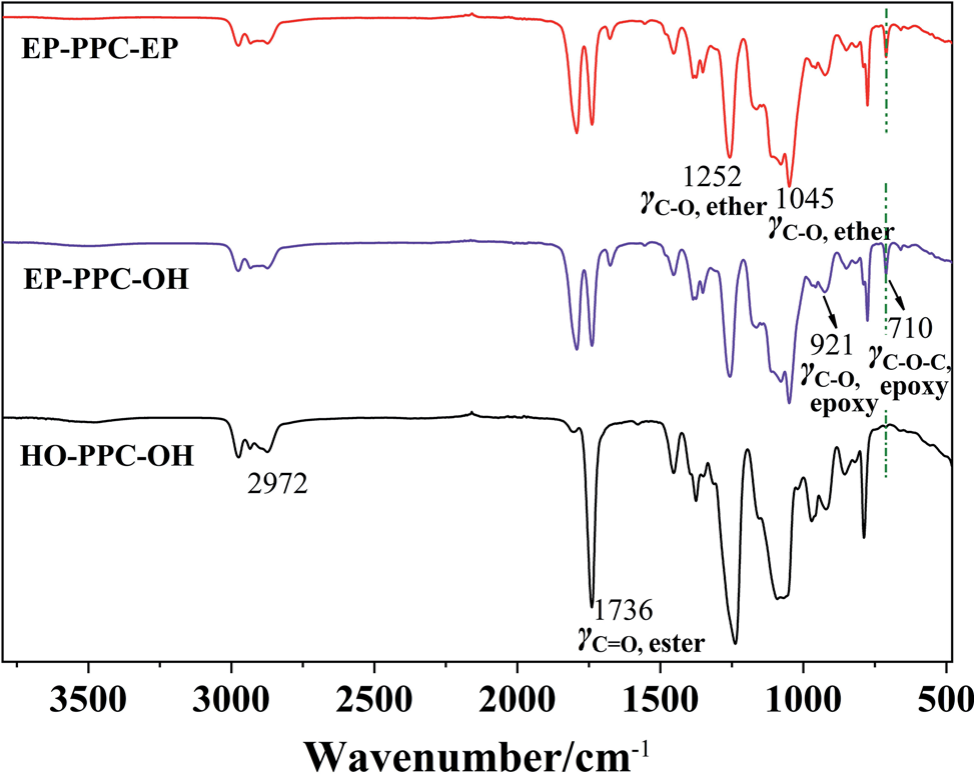
FTIR spectra of HO-PPC-OH, EP-PPC-OH and EP-PPC-EP.

The chemical structure and composition of the epoxy end-capped groups of the intermediate of EP-PPC-OH and EP-PPC-EP were ascertained by their proton NMR spectra (Fig. 1) and FTIR spectra (Fig. 2). The NMR peaks at *δ* = 4.85 ppm (a) and 4.10 ppm (b) were attributed to the methane (C***H***) and methylene (C***H***_2_) groups of the carbonate linkages of the PPC chain, which is from the copolymerization of the propylene oxide (PO) and CO_2_ monomers. The peak at *δ* = 3.50 ppm (a′, b′) was attributed to the ether linkages of the PPC chain which was from the homopolymerization of the PO monomer.^34^ The clear appearance of new peaks at *δ* = 2.82 and 2.64 ppm (f) were attributed to the epoxy ring protons, indicating that the epoxy groups were successfully capped on the end of the PPC chain. The integral area of peak (e) of epoxide at 2.82 ppm and peak (a) of PPC at 4.85 ppm can be used to determine the quantities of the mono- or bis-epoxy capped products. The presence of epoxy groups in the IR spectra (Fig. 2) is confirmed by the strong bands at 3056 cm^−1^ (*ν*_C–H_ epoxy), 921 cm^−1^ (*γ*_C–O_ epoxy) and 710 cm^−1^ (*γ*_C–O–C_ epoxy).

**Fig. 3:**
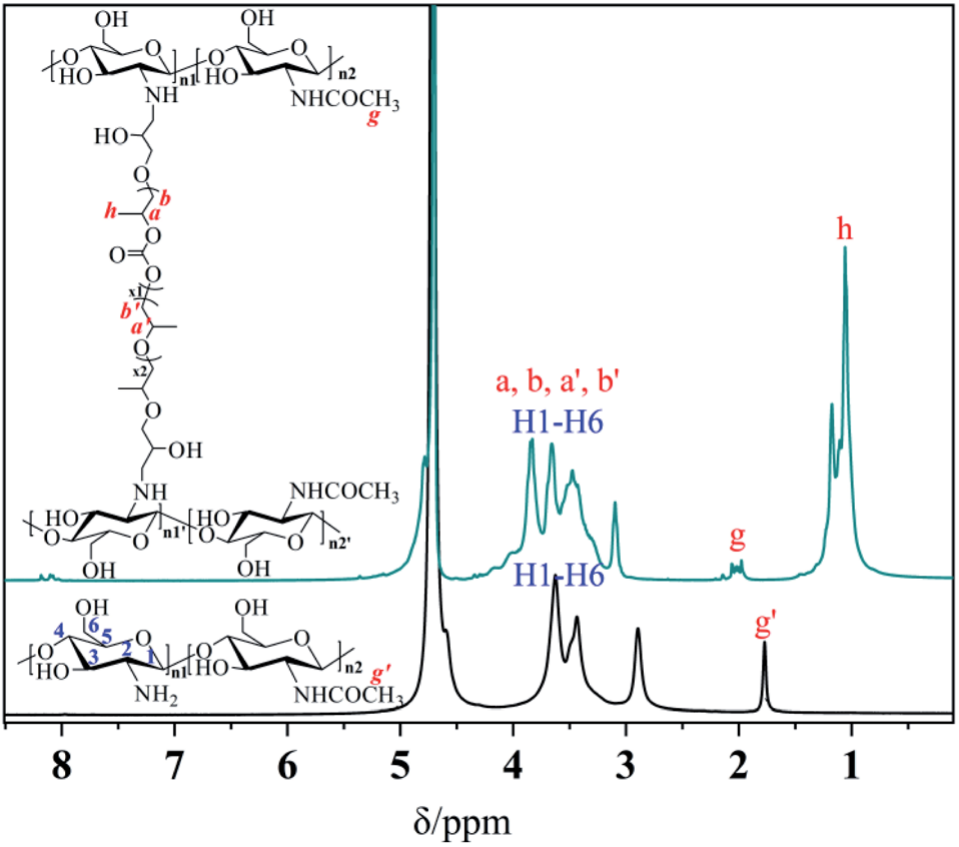
^1^H NMR spectra of chitosan and a selected CS-PPS microgel (in D_2_O, 500 MHz).

A close comparison between the proton NMR spectra of a typical CS/PPC sample and the water-soluble chitosan raw material is shown as an example in Fig. 3. The peak at *δ* = 1.95 ppm is the proton of the methyl of the *N*-acetyl group from the chitosan, while the peaks at *δ* = 3.60, 3.45 and 2.90 ppm are ascribed to the various protons of the glucose skeleton of chitosan.^35^ The appearance of new peaks at *δ* = 3.80 and 1.05 ppm correspond to the methyl, methylene and methane (a, b, a′, b′ and h) groups from PPC. The disappearance of the characteristic signals of the peaks at *δ* = 2.82 and 2.64 ppm (methylene and methane proton of the epoxide) strongly support the successfully synthesis of the target CS/PPC products.

**Fig. 4:**
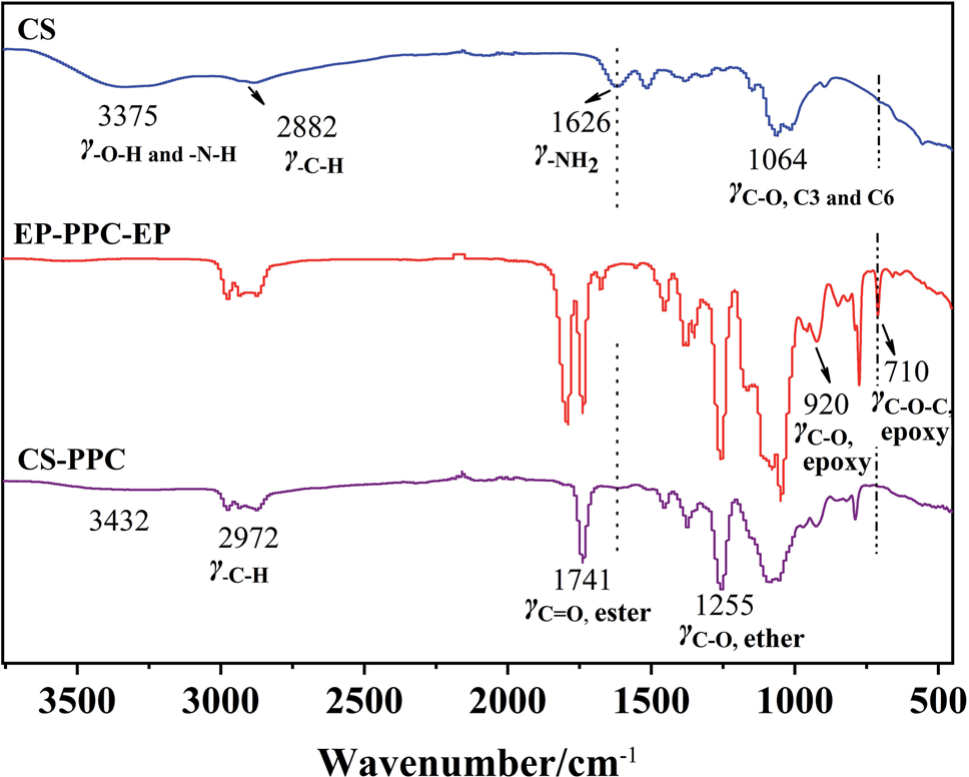
FTIR spectra of CS, EP-PPC-EP and selected CS-PPC nanoparticles.

A further comparison of the FTIR spectra of CS, EP-PPC and CS/PPC are shown as another example in Fig. 4. Chitosan exhibits main characteristic absorption bands of the amine group (–NH_2_) at 1626 cm^−1^. The broad band is consistent with the stretching vibration of –NH_2_ and –OH groups can be observed at 3000–3500 cm^−1^, and the peaks in the region 3000–2800 cm^−1^ is a result of C–H stretching. The peak at 2882 cm^−1^ is typical C–H vibration. The bands at 1000–1200 cm^−1^ are attributed to the saccharide structure of chitosan.^36^

Typical infrared (IR) peaks for PPC can be found by the characteristic stretching of carbonate C=O (1741 cm^−1^), and a peak of the O–C–O asymmetric stretching absorption band typically appears at 1290–1180 cm^−1^. The presence of the epoxy groups in the IR spectra can be found as strong absorption bands at 3056 cm^−1^ (*ν*_C–H_ epoxy) and 920 cm^−1^ (*γ*_C–O_ epoxy). As can be seen from the IR spectra of CS/PPC, the appearance of characteristic peaks of CS and PPC, the disappearance of the peaks of epoxy at 920 cm^−1^ and 710 cm^−1^, and the decreased absorbance peak of amine (–NH_2_) groups confirms that the end-capped epoxy groups of PPC have reacted through an epoxy-imine reaction with the amine group of CS.

### 3.3. Sizes, CMC, and zeta potential of CS/PPC nanoparticles

**Fig. 5:**
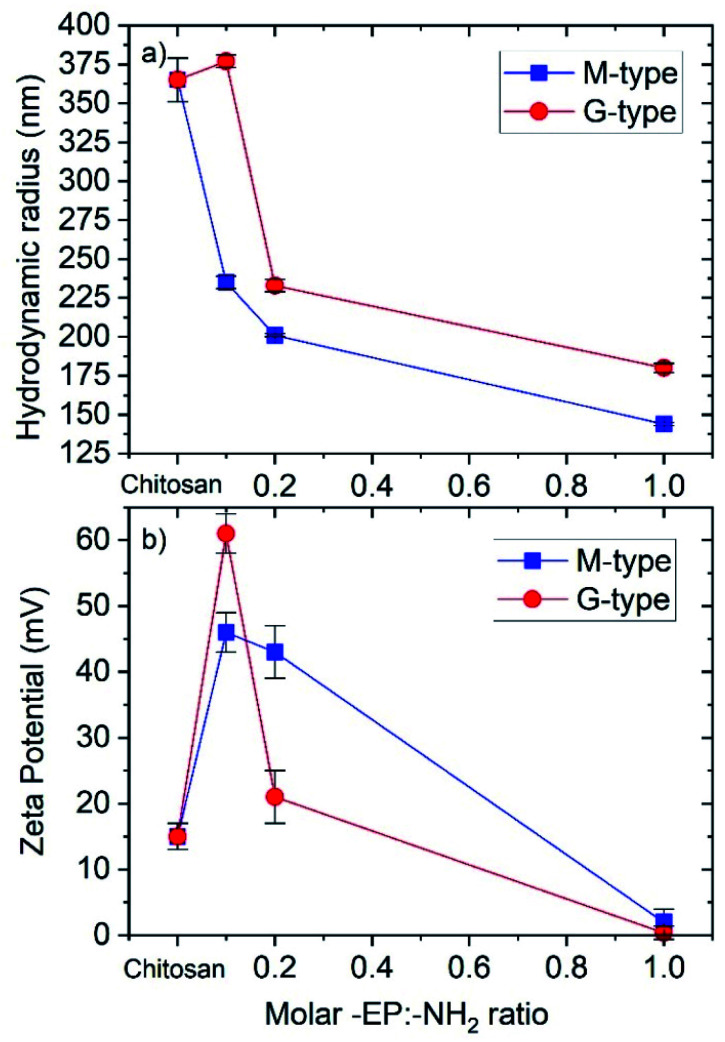
(a) Hydrodynamic radius and (b) zeta potentials of M-type and G-type NPs at a concentration of 1 mg mL^−1^. The polydispersity index (PDI) of the radius varied between 0.1 and 0.2.

The hydrodynamic radius of nanoparticles formulated by a series of CS-*g*-PPC (M-type NP) and CS–PPC microgels (G-type NP) in distilled water (1.0 mg mL^−1^) were measured by dynamic light scattering (DLS), and the results are shown in Fig. 5a. The average particle size of both M-type and G-type NP decreased as the amount of hydrophobic PPC units was raised. For the M-type NP (CS-*g*-PPC), the declining sizes are probably due to the formation of more compact spherical micelles when the hydrophobicity becomes higher. The CS–PPC microgels (G-type NP) form a more compact 3D structure at higher amounts of the cross-linking agent (EP-PPC-EP), thereby reducing the size of nanoparticles. It should be noted that one of the M-type samples M3 (235 nm) has the same size as the G-type sample G4 (233 nm). These two samples have the same total chemical composition (CS–PPC microgel, [EP-PPC-EP]/[NH_2_] = 0.2; CS-*g*-PPC, [EP-PPC-OH]/[NH_2_] = 0.1). Accordingly, they have the same hydrophilic/hydrophobic ratio, and can form hydrophobic cores of similar sizes, resulting in nanoparticles with the same size.

Zeta potential (surface charge) can greatly influence the stability of suspensions through electrostatic repulsion between particles. It can also influence the *in vivo* interactions between the nanoparticles and the cell membrane of bacteria. The zeta potentials of M-type and G-type NPs are shown in Fig. 5b. As expected, the pure chitosan samples have a positive zeta potential, due to positively charged NH_3_^+^-groups. Interestingly, the samples with low -EP/-NH_2_ feed ratios have higher zeta potentials than the samples with high -EP/-NH_2_ feed ratios and the pure chitosan. This is caused by the structure of the micelles and microgels, where the charged groups (which are hydrophilic) are pushed away from the hydrophobic cores of the NPs and out towards the surface of the NPs. When the -EP/-NH_2_ feed ratio is raised, the –NH_2_ groups are used up in the reaction, and fewer of these groups are available for protonation into –NH_3_^+^. Accordingly, the zeta potential decreases as the -EP : -NH_2_ feed ratio becomes higher.

**Fig. 6:**
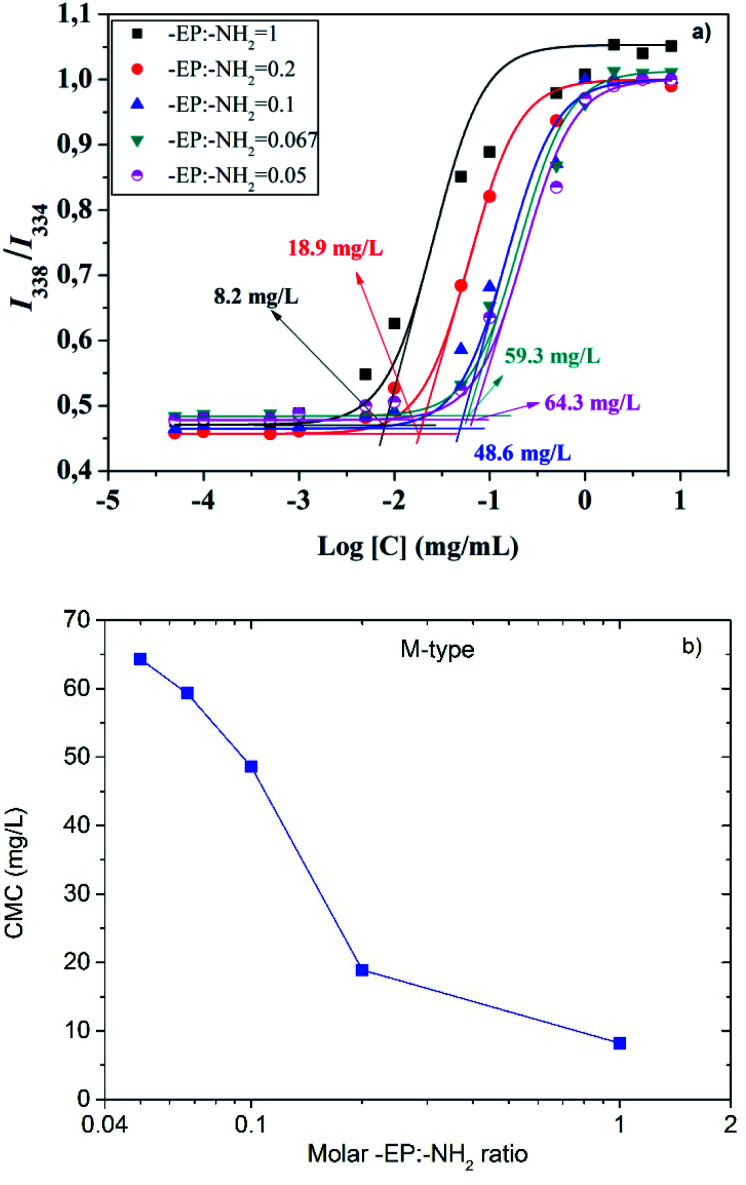
(a) Plot of the fluorescence intensity ratio *I*_338_/*I*_334_ obtained from excitation spectra of pyrene as a function of the M-type NP concentrations. (b) CMC of the M-type NP as a function of the -EP/-NH_2_ molar feed ratio.

The CS-*g*-PPC polymers have a hydrophilic CS backbone grafted with hydrophobic PPC side chains (Scheme 2). Amphiphilic CS-*g*-PPC polymers have the tendency to self-assemble to form nano-sized colloidal micelles in water at a concentration greater than the critical micelle concentration (CMC). These self-assemblies are oriented in such a way that the hydrophobic part (PPC) of the amphiphilic is kept in the core and the hydrophilic part (CS) is in contact with the water. CMC for CS-*g*-PPC polymers were determined by fluorescence intensity at emission bands *I*_338_/*I*_334_ by using pyrene as a hydrophobic probe for micelle formation,^37^ as illustrated in Fig. 6a. The CMC values decreased from 64.3 to 8.2 mg L^−1^ as the -EP : -NH_2_ ratio increased from 0.05 to 1 (Fig. 6b). A higher ratio results in an enhanced hydrophobicity, which is expected to lower the CMC.^38^

### 3.4. Antibacterial activities and hemolysis of CS/PPC-based polymers nanoparticles

The antibacterial activity of chitosan/PPC nanoparticles was probed against two representative clinically relevant bacterial strains, namely *E. coli* (Gram-negative) and *S. aureus* (Gram-positive). By employing a broth microdilution assay, MICs were taken at the lowest polymer concentrations required for the inhibition of bacterial growth after 24 h incubation. MBC (the lowest concentration of an antibacterial agent required to kill a bacterium over 24 hours) was also determined. Hemolytic toxicity was taken as the concentration causing 50% hemolysis of mouse red blood cells (HC_50_). The selectivity of the CS/PPC nanoparticles was calculated as the ratio between HC_50_ and MIC. The results of these experiments are collected in Table 1.

Interestingly, the NPs displayed significant antimicrobial activity against both Gram-negative bacteria (*E. coli*) and Gram-positive bacteria (*S. aureus*) even in neutral solutions. The MIC ranged from 128 μg mL^−1^ to 1024 μg mL^−1^, strongly dependent on the chemical composition of the nanoparticles (feed ratio of the PPC/CS, and the type of the reactive PPC (mono- or bis-epoxy capped)). The anti-microbial properties were found to be stronger against *E. coli* than *S. aureus*. In contrast, previous studies of water soluble *N*-modified chitosan derivatives did not show antimicrobial activity against neither *E. coli* or *S. aureus* at physiological pH 7.4.^39,40^

Chitosan and its derivatives exhibit different activity toward the Gram-positive and Gram-negative bacteria. In Gram-positive bacteria, the cell wall is made up of a thick peptidoglycan layer where negatively charged teichoic acids are covalently linked to *N*-acetylmuramic acid. While in Gram-negative bacteria, a thin peptidoglycan layer above the cytoplasmic membrane is further covered by an additional outer envelope called the outer membrane.^41^ Hydrophobic compounds and macromolecules are usually not active toward Gram-negative bacteria, and in order to interact with the Gram-negative bacteria, it is therefore essential to overcome the outer membrane barrier.^42^ Four mechanisms have been proposed to explain how chitosan interacts with the Gram-negative bacteria surface (either cell wall or outer membrane) causing its antimicrobial activity: (a) electrostatic interaction between the positively charged chitosan and the negatively charged residues on the bacteria surface; chitosan interacts with the membrane of the bacteria to alter cell permeability; (b) the interaction of hydrolysis products with microbial DNA, which leads to the inhibition of the mRNA and protein synthesis; (c) chitosan inhibits the microbial growth by chelation of nutrients and essential metals; (d) chitosan can form a polymer membrane on the surface of the cell, which prevents nutrients from entering the cell or acts as an oxygen barrier which can inhibit the growth of aerobic bacteria.^43^

Chitosan generally exhibits stronger bactericidal effects for Gram-positive bacteria than for Gram-negative bacteria in the presence of 0.1% chitosan acid solution, as observed by Jeon *et al.*^44^ As all samples in this study were prepared and evaluated for their activity in neutral medium, where the pH is higher than p*K*_a_ (6.5). Accordingly, significant protonation is not observed. Hydrophobic interaction and chelation effects result in antimicrobial activity of the chitosan derivatives. These two effects provide a reasonable explanation for the higher activity of chitosan derivatives under neutral or high pH than is observed for unmodified chitosan. The raw chitosan used in this study was water soluble and with a low viscosity of 8 mPa s. The viscosity average *M*_w_ is estimated to be 8.3 kDa by the Mark–Houwink equation: [*η*] = *KM*_w_^*a*^, where *K* and *a* is 1.81 × 10^−3^ cm^3^ g^−1^ and 0.93, respectively.^45^ Low-molecular-weight chitosan and its derivatives penetrate more easily into the cell wall of *E. coli*, and interfere with the metabolism of the bacteria and cause their death. However, inhibiting the growth of *S. aureus* requires the formation of a dense film on the cell surface, so low molecular weight chitosan has a weaker inhibition of *S. aureus* growth.

**Fig. 7:**
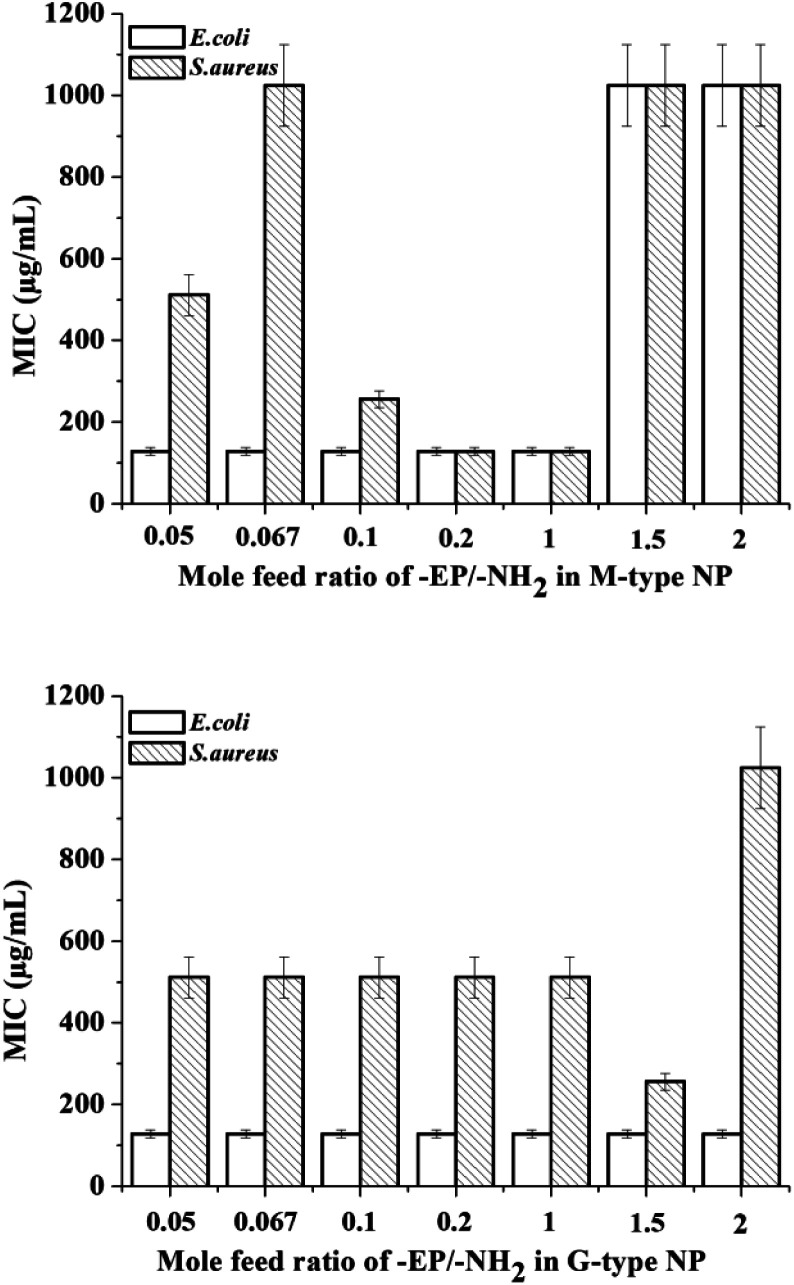
Antibacterial (MIC) activities of nanoparticles prepared at different feed ratios of -EP/-NH_2_.

As can be seen from Table 1 and Fig. 7, the M-type samples M1–M5 (prepared with [-EP]/[-NH_2_] ≤ 1) exhibited higher antimicrobial activity than M6–M7 (prepared with [-EP]/[-NH_2_] > 1). This series of polymers can be considered as grafting hydrophobic PPC to the main chain of the hydrophilic chitosan. The hydrophilic/hydrophobic balances influence the binding affinity of the nanoparticles on the surface of the bacteria (*S. aureus*). The hydrophobic modification of chitosan can favor the interaction of the polymer with the bacterial cells. The samples with highest hydrophilicity (M1, M2) is not effective for inhibiting *S. aureus* growth, indicating that the hydrophobic interaction and chelation effects are the two main factors determining the antimicrobial activity of chitosan derivatives in neutral medium. M6 and M7 showed poor antibacterial activity against both bacteria (*E. coli* and *S. aureus*), this is possibly due to their high hydrophobicity, which result in low solubility in water.

The G-type samples can be considered as hydrophilic chitosan crosslinked with hydrophobic PPC. The hydrophobic PPC chains are enclosed by the hydrophilic chitosan chains when they are prepared with a feed ratio [-EP]/[-NH_2_] ≤ 2. The overall hydrophobicity of the microgels increase at higher crosslinker contents. All the G-type nanoparticles (G1–G7) exhibit the same level of inhibition of *E. coli* (MIC = 128 μg mL^−1^). G1–G5 with low to medium hydrophobicity (0 < [-EP]/[-NH_2_] ≤ 1) have nearly the same inhibition ability to *S. aureus* (MIC = 512 μg mL^−1^), and G6 ([-EP]/[-NH_2_] = 1.5) reached the highest inhibition ability to *S. aureus* (MIC = 256 μg mL^−1^) and G7 ([-EP]/[-NH_2_] = 2) displayed the lowest inhibition ability to *S. aureus* (MIC = 1024 μg mL^−1^).

**Fig. 8:**
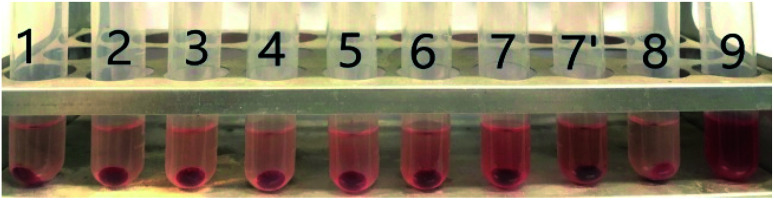
Photograph of the hemolytic test results of nanoparticles; tube 1–7 are G-type NP (G1, G2, G3, G4, G5, G6 and G7); tube 7′ is M7 (M-type sample of the same chemical composition as sample G7); tube 8 is the negative control, tube 9 is the positive control; [c] = 2048 μg mL^−1^.

Hemolysis is an important parameter for evaluating the safety of biological materials. The hemolytic activity is driven by the hydrophobic nature of the polymer. The polymers are expected to bind selectively to bacterial cell surfaces over human cells, because of electrostatic interactions. However, when the hydrophobicity dominates in the structure of the polymers, the polymers start to bind nonspecifically to both cell-types and enhance the permeability of the lipid bilayers in blood cells to rupture cell membrane and cause hemolysis.^46^ All the M-type and G-type nanoparticles were investigated against mouse red blood cells to evaluate their hemolytic activity at a concentration of 2028 μg mL^−1^ (see Table 1 and Fig. 8). No hemolysis was observed at this concentration, which is well in excess of the effective bacteria-inhibiting concentration. Selectivity was calculated using the hemolytic activity and the MIC for a given bacterium (*i.e.*, by dividing the HC_50_ value by the MIC, HC_50_/MIC). These values are given in Table 1. The highest selectivity (16) was found for M4 (prepared with [-EP]/[-NH_2_] = 0.2) and M5 (prepared with [-EP]/[-NH_2_] = 1.0) for both *E. coli* and *S. aureus*. This illustrates that all these PPC/CS nanoparticles have good biocompatibility. This is because the hydrophilic chitosan reduces the adsorption of red blood cells and further enhances its protective effect. Furthermore, the data also demonstrates that PPC can meet the biological safety assessment standards of GB/T16886 biomedical materials.^47^ Since the nanoparticles prepared from polypropylene carbonate and chitosan shows no hemolysis, they are expected have excellent prospects for biomedical applications.

## 4. Conclusion

A convenient manufactory process of biocompatible intrinsic antimicrobial chitosan nanoparticles was successfully developed by the reaction of an aqueous solution of commercial chitosan with synthetic mono-/bis-epoxy capped polypropylene carbonates. FTIR and proton NMR spectra confirmed successful formation of the epoxy-imine network between the amino groups of chitosan and the epoxide group of PPC. DLS shows that the mean size of both M-type and G-type nanoparticles decreased as the hydrophobic units of PPC increased due to the enhancement of the hydrophobicity of the polymers/nanoparticles. Interestingly, both CS/PPC nanoparticles (M-type and G-type) exhibited high antimicrobial activity (MIC = 128 μg mL^−1^) against *E. coli* and *S. aureus* and displayed no hemolytic activity to mouse red blood cells (HC_50_ > 2048 μg mL^−1^) in neutral medium, while the unmodified chitosan usually lose its inherent antibacterial activity in neutral medium. The work presented here suggests that CS/PPC-based nanoparticles exhibit a high potential for applications in biomedicine. This is of great significance for the research and development of new oligo-CO_2_-based antibacterial polymers of high value. Since the PPC is synthesized from CO_2_, this may also contribute to reduction of greenhouse gas emissions.

## Conflicts of interest

There are no conflicts to declare.

## Supplementary Material

RA-011-D0RA09257F-s001
