# Supplementary material for: Synthesis and antimicrobial activities of chitosan/polypropylene carbonate-based nanoparticles
Source: RSC Adv. 2021 Mar 10;11(17):10121–9. doi: 10.1039/d0ra09257f (PMC8695696; doi:10.1039/d0ra09257f)
Supplement: RA-011-D0RA09257F-s001 [file RA-011-D0RA09257F-s001.pdf]

Electronic Supplementary Material (ESI) for RSC Advances.
This journal is © The Royal Society of Chemistry 2021

Synthesis and Antimicrobial Activities of Chitosan/Polypropylene Carbonate-based Nanoparticles

Zhilong Quan[1], Chunyang Luo[1], Bitong Zhu[2], Chungui Zhao[2], Mingyi Yang[3], Magnar Bjørås [3], Kaizheng Zhu[4], Anna-Lena Kjøniksen[4*]*

[1]*College of Materials Science & Engineering, Huaqiao University, 361021 Xiamen, P.R China*

[2]*College of Chemical Engineering, Huaqiao University, 361021 Xiamen, P.R China*

[3]*Department of Microbiology, Oslo University Hospital, P.O. Box 4950, N-0424 Oslo, Norway*

[4]*Faculty of Engineering, Østfold University of College, P.O. Box 700, N-1757, Halden, Norway*

***Corresponding Authors**

Email: zlquan@hqu.edu.cn or anna.l.kjoniksen@hiof.no

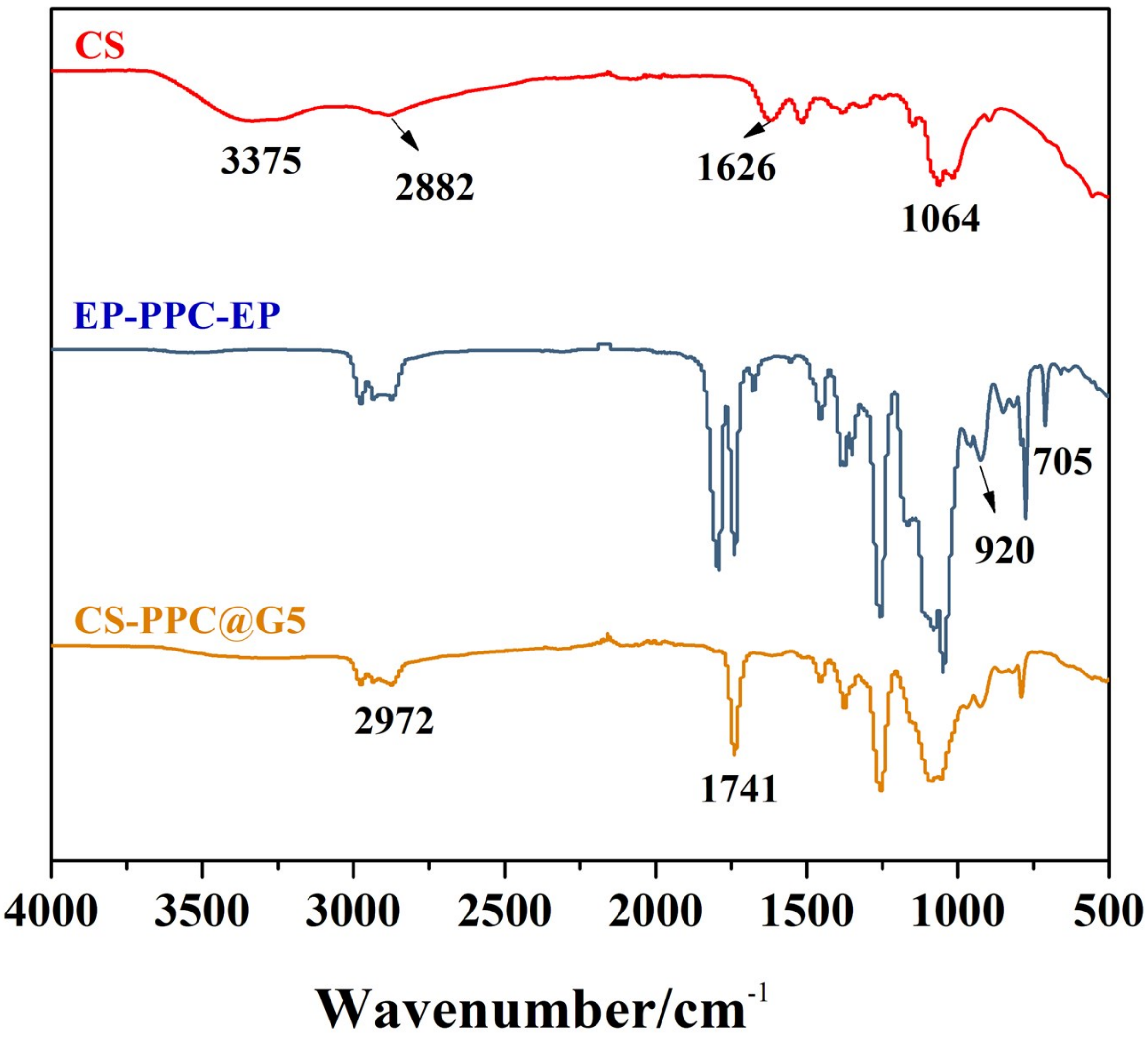

Fig. S1. FTIR Spectra of CS, EP-PPC-EP and G5

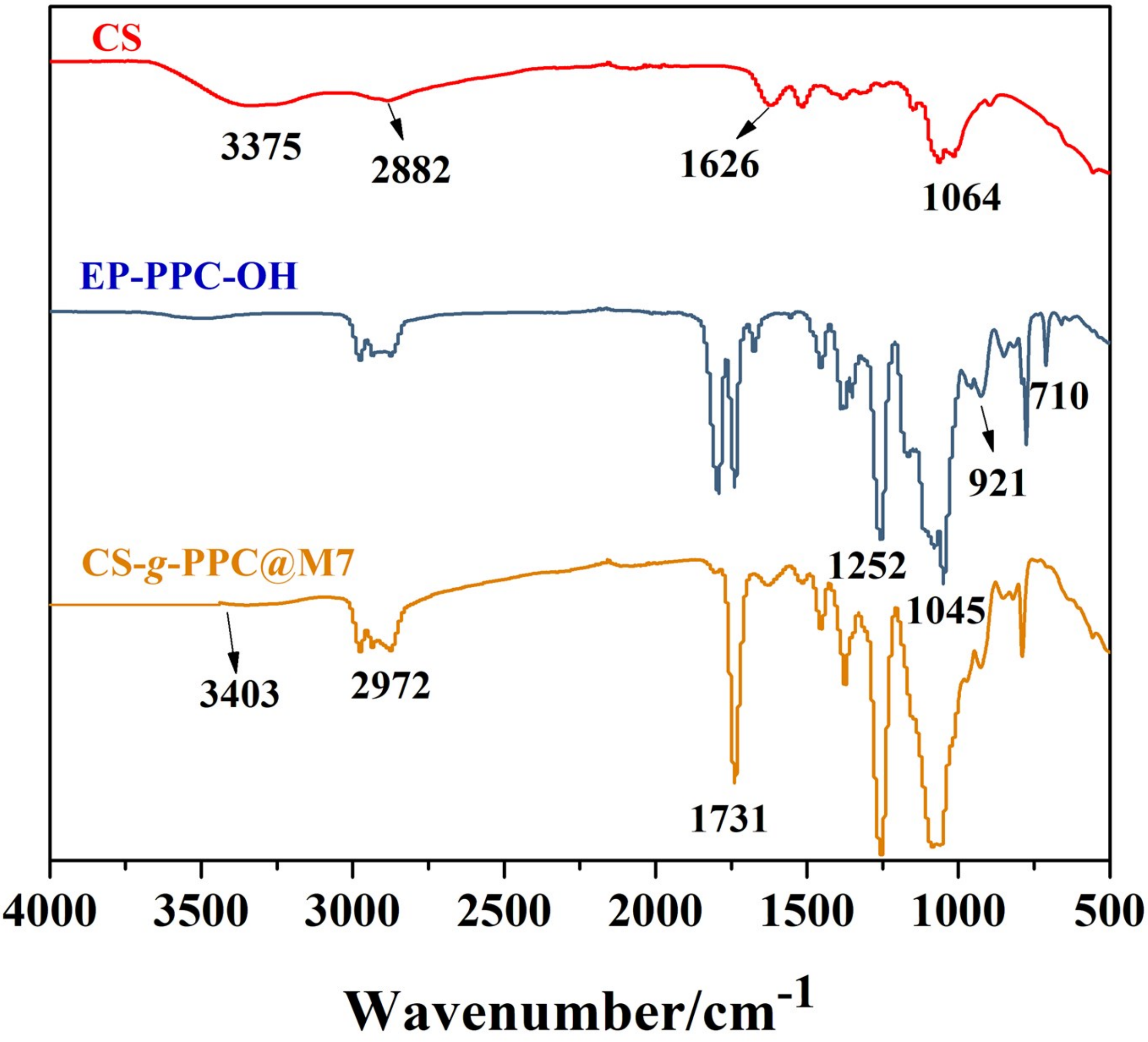

Fig. S2 FTIR of CS, EP-PPC-OH and M7

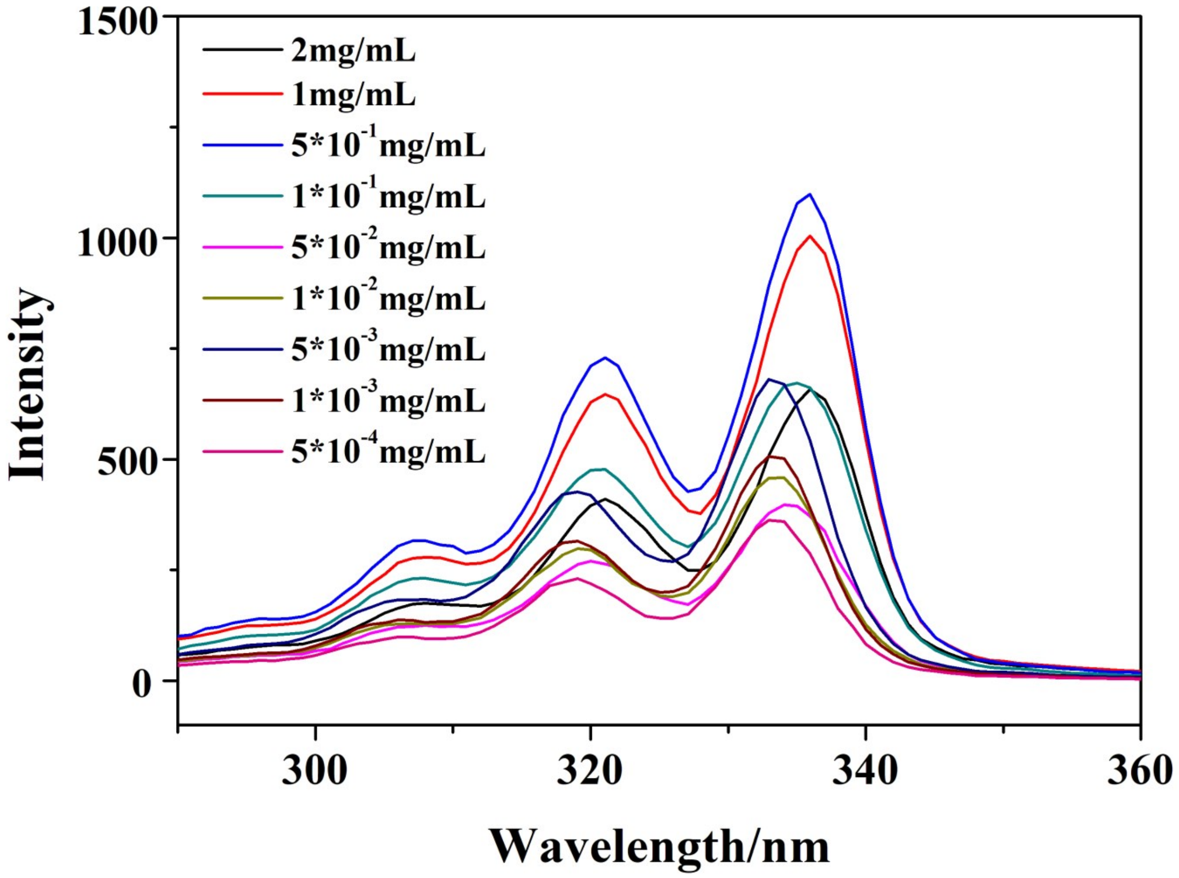

Fig. S3 PL of CS-*g*-PPC ([-EP]/[-NH_2] = 0.1) in different concentration [Pyrene]= 6.1×10^{-7} M

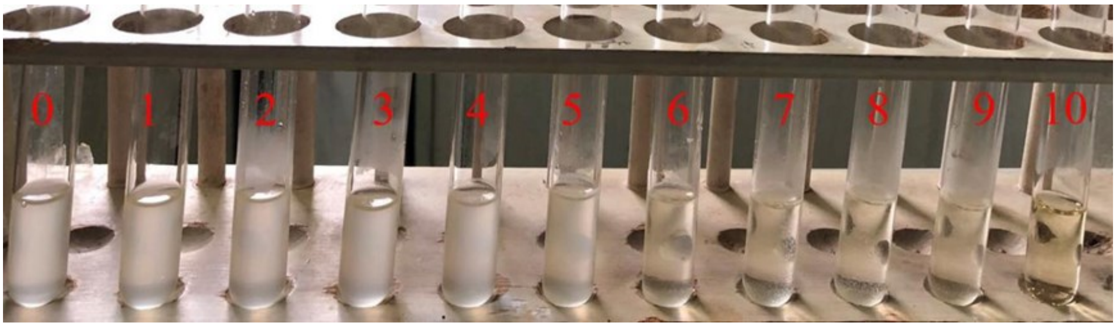

Fig. S4 Photograph of the MIC result of sample M5 ([-EP][/[-NH_2] =1] against *S.aureus* by tube dilution (turbidimetric) method：where: tube-0 is positive control, tube-10 is the negative control, tube 1~10 are samples of concentration 4 μg/mL, 8 μg/mL, 16 μg/mL, 32 μg/mL, 64 μg/mL, 128 μg/mL, 256 μg/mL, 512 μg/mL, 1024 μg/mL and 2048μg/mL, respectively.

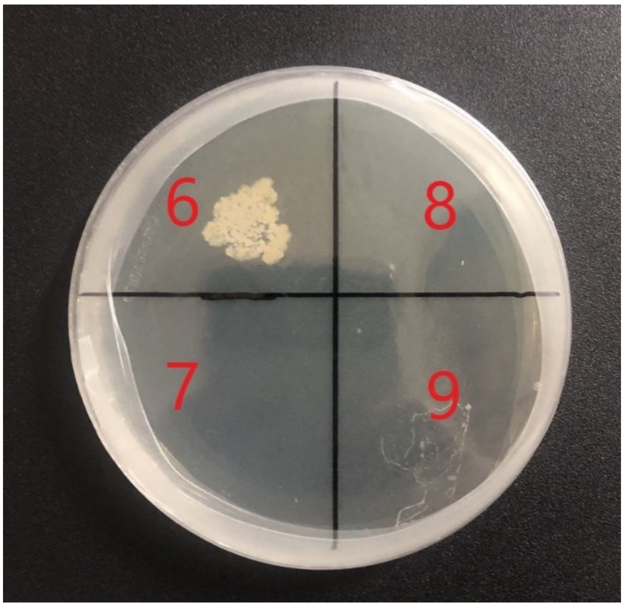

Fig. S5 Photograph of the MBC result of sample M5 ([-EP][/[-NH_2] =1] against *S.aureus*. Where 6~9 are sub-culture on agar plates with *S.aureus* treated with polymer samples of concentration 128 μg/mL, 256 μg/mL, 512 μg/mL and 1024 μg/mL, respectively.
